# Association of physical fitness with health-related quality of life in Finnish young men

**DOI:** 10.1186/1477-7525-8-15

**Published:** 2010-01-29

**Authors:** Arja Häkkinen, Marjo Rinne, Tommi Vasankari, Matti Santtila, Keijo Häkkinen, Heikki Kyröläinen

**Affiliations:** 1 Department of Physical Medicine and Rehabilitation, Central Hospital, Jyväskylä, Finland; 2 Department of Health Sciences, University of Jyväskylä, Jyväskylä, Finland; 3 UKK-Institute for Health Promotion Research, Tampere, Finland; 4 The National Institute for Health and Welfare, Helsinki, Finland; 5 Defence Command, Personnel Division, Finnish Defence Forces, Finland; 6 Department of Biology of Physical Activity, University of Jyväskylä, Jyväskylä, Finland

## Abstract

**Background:**

Currently, there is insufficient evidence available regarding the relationship between level of physical fitness and health-related quality of life (HRQoL) in younger adults. Therefore, the aim of the present study was to investigate the impact of measured cardiovascular and musculoskeletal physical fitness level on HRQoL in Finnish young men.

**Methods:**

In a cross-sectional study, we collected data regarding the physical fitness index, including aerobic endurance and muscle fitness, leisure-time physical activity (LTPA), body composition, health, and HRQoL (RAND 36) for 727 men [mean (SD) age 25 (5) years]. Associations between HRQoL and the explanatory parameters were analyzed using the logistic regression analysis model.

**Results:**

Of the 727 participants who took part in the study, 45% were in the poor category of the physical fitness, while 37% and 18% were in the satisfactory and good fitness categories, respectively. A higher frequency of LTPA was associated with higher fitness (*p *< 0.001). Better HRQoL in terms of general health, physical functioning, mental health, and vitality were associated with better physical fitness. When the HRQoL of the study participants were compared with that of the age- and gender-weighted Finnish general population, both the good and satisfactory fitness groups had higher HRQoL in all areas other than bodily pain. In a regression analysis, higher LTPA was associated with three dimensions of HRQoL, higher physical fitness with two, and lower number of morbidities with all dimensions, while the effect of age was contradictory.

**Conclusions:**

Our study of Finnish young men indicates that higher physical fitness and leisure-time physical activity level promotes certain dimensions of HRQoL, while morbidities impair them all. The results highlight the importance of health related physical fitness while promoting HRQoL.

## Background

The sedentary lifestyle presents a major public health challenge that must be met in order to prevent obesity and thus enhance health and well-being [1]. For substantial health benefits, current guidelines for adults recommend at least 2.5 hours of moderate-intensity or 1.25 hours of vigorous-intensity aerobic physical activity per week. Futher, moderate- or high-intensity muscle-strengthening activities for all major muscle groups two or more days a week provide additional health benefits [2]. According to the 2005 Eurobarometer on Health and Food, 41% of adults in EU-15 countries reported no moderate level physical activity in the past week and over half (59%) of the EU population get little or no physical activity at work [3]. The decrease in occupational and commuting physical activities should be compensated by an increase in LTPA as there is strong evidence regarding the protective effects of regular LTPA and a high level of physical fitness against major chronic diseases such as coronary heart disease, hypertension, stroke, noninsulin-dependent diabetes mellitus, osteoporosis, depression, and anxiety among others [4-7].

A systematic review has reported a consistent association of higher health-related quality of life (HRQoL) scores with higher PA levels among healthy adults [1]. Physical activity has enhanced well-being and increasing physical functioning also in people with poor health [8] or of advanced age [9]. Also higher physical fitness level has been shown to be associated with higher levels of HRQoL in the older and chronically diseased populations [10-12]. However, there is insufficient evidence regarding the relationship between physical fitness level and HRQoL in younger adults. One recent study has reported associations between cardiorespiratory fitness and HRQoL in young males in United States navy. They found a positive relationship between submaximal fitness test and mental and physical components of HRQoL [13]. There is still limited evidence on relationships of objectively measured fitness and individual domains of HRQoL. Therefore, the aim of the present study was to investigate the impact of measured cardiovascular and musculoskeletal physical fitness level on HRQoL in Finnish young men.

## Methods

The study participants were enrolled from April 2008 to November 2008 during eighth refresher course organized in different counties around the country; thus, they geographically represent the entire country. Of 1,155 invited reservists, 922 participated in the courses and 845 men volunteered for the present study. During the analysis phase, a further 118 participants were excluded because they had missed physical fitness tests (if any of the endurance or muscle fitness test results were missing, the physical fitness index [PFI] could not be calculated). Thus, the final study group consisted of 727 men with mean (SD) age of 25 (5) years. The participants signed a written consent form indicating that they were aware of the risks and benefits of the study. The study was approved by the ethical committees of the University of Jyväskylä and the Central Finland Health Care District, as well as the Headquarters of the Finnish Defence Forces.

### Measurements

#### HRQoL

In public health and in medicine, the concept of health-related quality of life refers to a person's or group's perceived physical and mental health over time. In this study HRQoL data were collected using the Finnish Rand 36-item health survey 1.0, which was developed from the original 36-Item Short Form Health Survey (SF-36) [14]. RAND-36 measures eight dimensions: general health, physical functioning, role limitation physical, role limitation emotional, vitality, mental health, social functioning, and bodily pain. There is a 0-100 range in each subscale, with higher scores indicating higher HRQoL. The reliability and validity of the scale has reported to be good (Cronbach's alpha coefficients for 8 dimensions varied between 0.80 and 0.94), but ceiling effects were detected for physical functioning, role limitation physical and social functioning dimensions and floor effect for role limitation physical, role limitation emotional dimensions [14]. The age- and sex-weighted Finnish general population was used as a reference study group [14].

#### Physical fitness index (PFI)

Oxygen uptake (VO_2_max) was indirectly measured using a bicycle ergometer test (Ergoline 800 S, Ergoselect 100 K or 200 K, Bitz, Germany) [15]. The handlebars and seats were individually adjusted. After a 5-min warm up, the test began with a power output of 75 W, which was increased by 25 W after every other minute. The pedalling rate of 60 rpm was maintained throughout the test. The heart rate (HR) was recorded continuously (Polar Vantage NV or S610, S710 or S810, Kempele, Finland). The test was terminated at volitional exhaustion, including a decrease in the pedalling rate to below 50 rpm. Predicted VO_2_max was determined from the HR and power (Fitware, Mikkeli, Finland), as follows: VO_2_max (ml·kg^-1^·min^-1^) = [(P_max _* 12.48) + 217]/body mass, where Pmax is maximal power. The test-retest repeatability was r = 0.89 and 0.96 for women and men, respectively [16].

Muscle fitness was measured by four consecutive tests: grip strength, push-ups, sit-ups, and repeated squats [14]. Before testing commenced, supervisors demonstrated the technically correct way to perform each test; they also controlled the performance technique of each person. Isometric grip strength was measured in a sitting position (90° elbow angle) by a dynamometer (Saehan Corporation, Masan, South Korea). The test was repeated twice separately for both hands; the best results for the right and left hands were averaged for the outcome [17]. Sit-ups, which measure performance of abdominal and hip-flexor muscles, were done with each subject lying supine on the floor with his hands behind the neck and directing his elbows forward. The knees were flexed at an angle of 90°, the legs were slightly apart, and the assistant supported the ankles. During the movement, the each subject lifted his upper body and touched his elbows to the knees. Push-ups, which measure performance of arm- and shoulder-extensor muscles, were started from the lowest face-down position. Each subject's hands were kept shoulder-wide and level. The fingers were directed forward, and the legs were kept parallel and close to each other. During the movement, the arms were fully extended and the torso was straightly tensed. In the second phase, the torso was lowered down to an elbow angle of 90°. Repetitive squats measure the strength of the knee extensors. The subject was standing with feet just inside shoulder width apart and squat was performed until the thighs were horizontal. The results of the push-ups, sit-ups, and repeated squats were expressed as the number of correctly performed repetitions within 60 s. The recovery time between each of the tests was 5-10 min.

In PFI calculations the absolute results for each muscle fitness test were scored to corresponding fitness categories from poor (1.0-1.9) to excellent (5.0-5.9). The total muscle fitness index was the sum of 4 muscle fitness tests. Finally PFI was determined utilizing an adjusted nomogram" where aerobic fitness and muscle fitness are equally important (50 and 50%). Accordingly, the PFI also had five different categories: excellent (5.0-5.9), good (4.0-4.9), satisfactory (3.0-3.9), fair (2.0-2.9), and poor (1.0-1.9). For statistical analyses, the PFI was categorized as poor (combination of categories fair and poor), satisfactory, or good (good and excellent) [17]. The reference values are based on the results of 3635 civilians and include 5 year age-specific categories [18]. These VO2max and muscle fitness tests have been used during this past decade (2000-2009) in the Finnish Defense Forces in order to follow-up the fitness components of professional soldiers and reservists and, in addition, to find out the general population based trends in fitness changes.

#### LTPA

The frequency and intensity of weekly LTPA was determined from responses to a single question with six categories: (1) no physical activity at all, (2) some physical activity without feeling out of breath or sweating, (3) physical activity with feeling out of breath or sweating once a week, (4) physical activity with feeling out of breath or sweating twice a week, (5) physical activity with feeling out of breath or sweating three times a week, and (6) physical activity with feeling out of breath or sweating at least four times a week. In the analysis, the participants were recorded to three groups according their physical activity level: low (combination of LTPA categories 1 and 2), moderate (categories 3 and 4), or high (categories 5 and 6) [16].

#### Health examination

Height and weight were measured while the participants were wearing lightweight clothing. Body mass index (BMI) was classified in five categories: severe obesity, ≥35.0; obesity, 30.0-34.9; overweight, 25.0-29.9; normal 19.0-24.9; and underweight, ≤18.9. Body fat and lean mass percentages were recorded using the eight-polar bioimpedance method with multifrequency current (InBody 720; Biospace Company, Seoul, Korea). Bioimpedance was performed in the postabsorbtive state after a 12-hour overnight fast and the day preceding the measurement day was a rest day from intensive exercise. For men the test-retest reliability of the device has shown to be high (ICC 0.9995) and no significant mean (SD) difference was found for body fat between two trials [20.98 (8.88)% and 21.00 (8.83)% [18].

Alcohol and tobacco product use was determined by a questionnaire. In addition, a number of self-reported morbidities that had been diagnosed by medical doctors were discovered by asking the respondents if they had pulmonary or heart disease, hypertension, inflammatory joint disease, or musculoskeletal disease. Self-perceived general health was assessed using a visual analogue scale, and self-perceived physical fitness compared to age mates was asked using five categories (highly lower, somewhat lower, equal, somewhat better, highly better).

### Statistics

The results are provided as means with standard deviation (SD) or 95% confidence level (CI). The normality of variables was evaluated by Kolmogorov-Smirnoff test and by means of histograms. The statistical significance of characteristics among the groups was evaluated by analysis of variance (ANOVA). If the variables did not fill normality assumptions, Kruskal-Wallis nonparametric test with appropriate pair-wise comparisons or chi-square test was used. The Finnish population values for the eight dimensions were weighted to match the age distribution of the study population. Associations between HRQoL and the explanatory parameters (age, LTPA, BMI, tobacco use, and morbidities) were analyzed using the logistic regression analysis model. Before regression analysis Spearman's Rank correlation coefficient was used to give an indication of the magnitude of association (collinearity) between explanatory variables and they were considered highly associated if their correlation coefficient was greater than 0.7.

## Results

When the participants were grouped according their objectively measured physical fitness indices (PFI) 45% of them belong to the poor, 37% to the satisfactory and 18% to the good fitness category. The mean (SD) PFIs were 2.44 (0.35), 3.43 (0.28), and 4.61 (0.47), respectively. The mean (SD) age of all of the participants was 25 (5) years (range 20-47). Mean (SD) BMI was 25 (4) (range 16.8-43.1); 60% of the participants had a normal BMI, 31% were overweight, and 9% were obese. Men in higher PFI categories had a lower BMI and a lower proportion of body fat (Table 1). The correlation between BMI and body fat was 0.81 (0.79 to 0.84). The lean body mass proportion did not differ among the PFI groups.

**Table 1:** Sample characteristics by physical fitness index.

Variable	Physical fitness index			P-value between the groups
		
	Poor (n = 328)	Satisfactory (n = 271)	Good (n = 128)	
Age in years, mean (SD)	25 (3)	25 (5)	27 (7)	0.29

Weight, kg, mean (SD)	85 (15)	78 (10)	73 (9)	< .0.001

Height, cm, mean (SD)	180 (6)	180 (6)	179 (6)	0.19

Body mass index, n (%)				< .0.001 *
<19	8 (3)	8 (3)	3 (2)	
19-24.9	138 (42)	173 (64)	106 (83)	
25-29.9	127 (39)	79 (29)	18 (14)	
≥30	53 (16)	11 (4)	1 (1)	

**Body fat, mean (SD)**	21.3 (6.9)	16.1 (5.7)	12.0 (4.5)	< 0.001

**Lean body mass, mean (SD)**	65.9 (8.2)	65.3 (7.1)	64.3 (6.6)	0.19

Alcohol users ≥ once a week, n (%)	219 (67)	175 (65)	74 (58)	0.20
Tobacco users, n (%)	161 (49)	85 (32)	20 (16)	< 0.001
Snuff users, n (%)	12 (4)	20 (7)	4 (3)	0.062

Self perceived general health, mean(SD)	25 (19)	21 (18)	18 (15)	< 0.001

Self-reported morbidities, n (%)	110 (33)	82 (30)	40 (31)	0.72 *

Self perceived physical fitness compared to age mates, n (%)				< 0.001 *
Highly lower	6 (2)	3 (1)	0(0)	
Somewhat lower	141 (43)	23 (9)	2 (1)	
Equal	152 (46)	128 (47)	41 (32)	
Somewhat better	27 (8)	96 (35)	61 (48)	
Highly better	2 (1)	21 (8)	24 (19)	

Self-reported leisure time physical activity, n (%)				< 0.001
Low	159 (48)	53 (20)	8 (6)	
Moderate	134 (41)	109 (40)	44 (34)	
High	36 (11)	108 (40)	76 (59)	

ANOVA or it's nonparametric equivalent Kruskall-Wallis-test

* Chi-square

The proportion of tobacco use increased with decreasing PFI. Self-perceived general health was lower in the poor PFI group. The number of other morbidities did not differ among the groups. The most commonly reported morbidities were musculoskeletal disease (*n *= 171), pulmonary or heart disease (*n *= 45), and hypertension (*n *= 34).

In the poor objectively measured PFI group, 45% of the participants graded their self-perceived physical fitness as lower compared to age mates, while 9% graded it as higher (Table 1). In the good PFI group, the respective proportions were 1% and 67%. A higher frequency of LTPA was associated with a higher PFI. The correlation between PFI and LTPA was 0.49 (95% CI 0.44-0.55).

**Figure 1:**
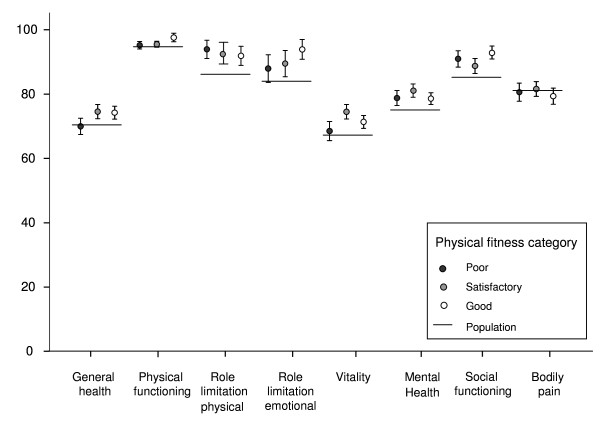
**Health-related quality of life dimensions (SF-36) of Finnish young men compared to age-matched male population**. (means with 95 percent confidence intervals). Line shows age adjusted values of general population.

A higher HRQoL score in the general health, physical functioning, vitality and mental health, dimensions was associated with a higher PFI (Table 2). When the HRQoL of the participants was compared with that of the age- and gender-weighted Finnish population both the good and the satisfactory PFI participants had a higher HRQoL than the general population in all of the dimensions except for bodily pain (Figure 1). In the poor physical fitness group, role limitation physical, mental health and social functioning dimensions were on a higher level compared to the general population.

**Table 2:** Health related quality of life (RAND-36) in 727 Finnish young men according to their physical fitness index (PFI)

	PFI-groups			P-value between the groups*
		
	Low Mean (95% Cl)	Satisfactory Mean (95% Cl)	High Mean (95% Cl)	
General health perception	70.0 (67.4 to 72.5)	74.5 (72.4 to 76.7)	74.2 (72.2 to 76.3)	< 0.001
Physical functioning	95.2 (94.0 to 96.3)	95.5 (94.6 to 96.4)	97.6 (96.3 to 98.9)	< 0.001
Role limitation physical	93.9 (91.1 to 96.8)	92.4 (89.1 to 95.8)	91.9 (88.9 to 94.7)	0.98
Role limitation emotional	87.9 (83.6 to 92.2)	89.5 (85.4 to 93.6)	93.9 (90.8 to 97.0)	0.78
Vitality	68.5 (65.6 to 71.5)	74.5 (72.2 to 76.7)	71.3 (69.3 to 73.3)	0.034
Mental health	78.8 (76.5 to 81.1)	81.179.0 to 83.1)	78.6 (76.8 to 80.4)	0.029
Social functioning	90.9 (88.4 to 93.5)	88.8 (86.4 to 91.1)	92.8 (90.9 to 94.7)	0.32
Bodily pain	80.6 (77.7 to 83.4)	81.6 (79.3 to 83.9)	79.3 (76.8 to 81.8)	0.35

Regression analysis revealed that a lower number of morbidities was related to a higher HRQoL in all eight dimensions (Table 3). Both higher PFI and LTPA were associated with general health and physical functioning and higher LTPA with the vitality dimension. Lower age was associated with better physical functioning, while higher age with better role limitation emotional, vitality, and mental health.

**Table 3:** Logistic regression analysis of eight HRQoL dimensions (RAND-36).

	General health perception	Physical functioning	Role limitation physical	Role limitation emotional	Vitality	Mental health	Social functioning	Bodily pain
Age	0.98 (0.94 to 1.01)	0.96(0.92 to 0.99)*	1.00(0.95 to 1.04)	1.05(1.00 to 1.10)*	1.08(1.03 to 1.12)*	1.05(1.01 to 1.09)*	1.02(0.98 to 1.06)	0.98(0.94 to 1.01)

PFI	1.63 (1.27 to 2.09)*	1.56(1.19 to 2.05)*	1.05(0.77 to 1.43)	1.09(0.84 to 1.42)	1.15(0.91 to 1.45)	1.01(0.80 to 1.28)	1.09(0.87 to 1.37)	1.09(0.86 to 1.39)

LTPA	1.27 (1.12 to 1.43)*	1.28(1.12 to 1.46)*	0.99(0.85 to 1.16)	1.03(0.90 to 1.17)	1.12(1.00 to 1.26)*	1.01(0.90 to 1.14)	1.05(0.93 to 1.17)	1.08(0.96 to 1.22)

BMI	1.01 (0.96 to 1.06)	0.98(0.93 to 1.03)	1.01(0.95 to 1.08)	1.01(0.96 to 1.07)	1.04(0.99 to 1.09)	1.02(0.97 to 1.07)	1.05(1.00 to 1.10)	1.00(0.96 to 1.06)

Morbidities	0.47 (0.33 to 0.65)*	0.30(0.21 to 0.43)*	0.39(0.26 to 0.59)*	0.60(0.42 to 0.85)*	0.61(0.44 to 0.84)*	0.62(0.45 to 0.86)*	0.56(0.41 to 0.77)*	0.24(0.17 to 0.34)*

Only those variables are shown which were entered into model.

## Discussion

Results of the present study showed in a relatively large sample of Finnish men that higher PFI was associated with more favorable scores in the general health, physical functioning, mental health, and vitality dimensions of HRQoL. The importance of PFI was supported by our finding that the good and satisfactory PFI groups had a higher HRQoL score in all of the dimensions except for bodily pain, compared to the reference values of the age- and gender-weighted Finnish population. The lack of difference in the bodily pain dimension may reflect the fact that the number of morbidities did not differ among the fitness categories. Previous studies have shown that cardiorespiratory fitness is associated with physical functioning in 40-65-year-old participants with diabetes [19] and 40-60-year-old Finnish men working in blue-collar occupations [6]. When we repeated regression analysis of our study group and entered VO_2_max and muscle fitness index separately in the model, instead of combined PFI, VO_2_max was associated with general health perception and muscle fitness index was associated with physical functioning and general health perception (data not shown). A recent study including healthy 18-49 years old men from United States navy showed a positive relationship between submaximal exercise test and mental and physical health components of HRQoL [13].

The results presented here provide support for earlier findings of cross-sectional studies, which showed that higher levels of LTPA were associated with certain HRQoL dimensions [20-22]. Vuillemin et al. (2005) reported that in men, LTPA was related to all of the other dimensions except for emotional role functioning [20]. Wendel-Vos et al. (2004) showed that meeting recommended levels of physical activity (at least 30 minutes of moderate LTPA per day) was associated with higher HRQoL scores in all dimension [22]. When interpreting our results and the results of others, it is important to note that some participants may under- or overestimate the intensity of their LTPA. In the present study, over half of the participants in the poor PFI category reported that their LTPA was moderate or high, while some of the participants in the good PFI category reported that it was low. Some respondents may not perceive their activity as sufficiently moderate or vigorous, and may have underestimated their LTPA level. Likewise, some respondents may have misreported their PA levels to reflect the socially desirable nature of PA participation; thus, they may have overestimated their LTPA level [23]. However, we found that both self-reported LTPA levels and measured PFIs were associated with the general health perception and physical functioning dimension. Our finding that LTPA was also associated with vitality dimension is supported by a review by Puetz (2006) showing that people who are physically active in their leisure time have about a 40% reduced risk of experiencing feelings of low energy and fatigue compared to sedentary people [24].

In the good PFI group, we found that the proportion of body fat was lower than in the low PFI group. However, the amount of lean body mass did not differ among the PFI groups, although BMI increased with decreasing PFI. BMI was not associated with HRQoL. Further, when the percentage of body fat was entered into the regression model instead of BMI, the only statistically significant association we found was that a high body fat percentage was associated with the better mental health dimension (data not shown). The findings of previous studies of the effect of body weight on HRQoL are controversial. Some studies have reported that obese adolescents have a poorer HRQoL than lean individuals [25]. On the other hand, in accordance with our results, other studies did not find a significant relationship between BMI and HRQoL [26]. These confounding results of different studies may be partly explained by differences in the gender, sample size, age, and range of BMI of the participants. Furthermore, it is possible for a healthy, well-trained muscular individual with very low body fat to be classified as obese using the BMI formula. However, higher body fatness and lower physical fitness has reported to be associated with metabolic risk factors even in late adolescent college students thus increasing the risk of chronic diseases later in life [27].

Morbidities were an important explanatory variable of the impairments found in all eight HRQoL dimensions. The diseases that were reported decreased the physical, mental, and social functioning of the participants. A previous study showed that musculoskeletal pain has a negative effect on the HRQoL of elderly people living in Turkey [28]. A German study found that general practice patients with chronic diseases had impaired quality of life, particularly with regard to physical health [29]. The independent effects of the morbidities on HRQoL varied depending upon the type of chronic disease: HRQoL appeared to be more affected by diseases such as depression, back pain, osteoarthritis of the knee, and cancer than by hypertension and diabetes [30]. Asymptomatic status and health risks such as hypertension or MBO were reported to be less likely to affect quality of life [29,31], while study participants were more conscious of and thus affected by physical medical symptoms leading to a discernable limitation in performance [32,33].

In Finland a universal male conscription is in place, under which all men above 18 years of age serve for 6, 9 or 12 months, these reservists which are invited to the refresher courses represent rather well Finnish young men. Some of the reservists from which we drew our study participants were unable to attend the courses because of personal or social reasons or health conditions, or because they were living abroad. Thus, a limitation of the present study is that we do not know the characteristics of those reservists who did not enter the courses. It was previously suggested that males may underestimate problems of functional capacity and pain on questionnaires [32,34], which may also have affected the data regarding our study participants. The range of the age was 20-47 years. Increasing age does not necessarily cause a reduction in the quality of life, but it may shift the emphasis of it as shown with the present data. Although 32% of the participants had self-reported morbidities, the ceiling effect was over 15% in 5 out of 8 dimensions of HRQoL (physical functioning, role limitation physical, role limitation emotional, social functioning and bodily pain). However, although this is a cross-sectional study, the strength of this study is that participants' subjective perspectives on physical fitness and health were accompanied by objective measurements of maximal aerobic capacity and muscle endurance, making the findings more accurate and dependable.

## Conclusions

The present study on Finnish young adult men showed that higher physical fitness and leisure-time physical activity level promotes certain dimensions of HRQoL, and the higher number of morbidities impairs all of them. Because physical fitness was associated with the young men's HRQoL and health and, thus, their value to the present and future labour force, feasible methods to promote PA levels and thereby HRQoL in young men should be pursued.

## Competing interests

The authors declare that they have no competing interests.

## Authors' contributions

The authors of this manuscript state that all of them have contributed substantially to manuscript preparation. All authors read and approved the final manuscript.
